# Sensory Reweighting for Postural Control in Older Adults with Age-Related Hearing Loss

**DOI:** 10.3390/brainsci13121623

**Published:** 2023-11-23

**Authors:** Lydia Behtani, Daniel Paromov, Karina Moïn-Darbari, Marie-Soleil Houde, Benoit Antoine Bacon, Maxime Maheu, Tony Leroux, François Champoux

**Affiliations:** 1 School of Speech Language Pathology and Audiology, Faculty of Medecine, Université de Montréal, Montréal, QC H3T 1J4, Canada; lydia.behtani@umontreal.ca (L.B.); daniel.paromov@umontreal.ca (D.P.); karina.moin-darbari@umontreal.ca (K.M.-D.); mariesoleilhoude@gmail.com (M.-S.H.); maxime.maheu1@umontreal.ca (M.M.); tony.leroux@umontreal.ca (T.L.); 2 Centre de Recherche de L’institut Universitaire de Gériatrie de Montréal (CRIUGM), Montréal, QC H3W 1W4, Canada; 3 Department of Psychology, Faculty of Art and Science, University of British Columbia, Vancouver, BC V6T 1Z4, Canada; benoit.antoine.bacon@gmail.com; 4 Institut Universitaire Sur la Réadaptation en Déficience Physique de Montréal (IURDPM), Pavillon Laurier, CIUSSS du Centre-Sud-de-l’Île-de-Montréal, Montréal, QC H2H 2N8, Canada

**Keywords:** age-related hearing loss, aging, audio–motor interaction, audio–tactile interaction, deafness, hearing loss, postural control, multisensory interaction, sensory reweighting

## Abstract

There is growing evidence linking hearing impairments and the deterioration of postural stability in older adults. To our knowledge, however, no study to date has investigated the effect of age-related hearing loss on the sensory reweighting process during postural control. In the absence of data, much is unknown about the possible mechanisms, both deleterious and compensatory, that could underly the deterioration of postural control following hearing loss in the elderly. The aim of this study was to empirically examine sensory reweighting for postural control in older adults with age-related hearing loss as compared to older adults with normal hearing. The center of pressure of all participants was recorded using a force platform and the modified clinical test of sensory interaction and balance protocol. The results suggest that individuals with age-related hearing loss displayed increased somatosensory reliance relative to normal hearing younger adults. This increased reliance on somatosensory input does not appear to be effective in mitigating the loss of postural control, probably due to the concomitant deterioration of tactile and proprioceptive sensitivity and acuity associated with aging. Beyond helping to further define the role of auditory perception in postural control, these results further the understanding of sensory-related mechanisms associated with postural instability in older adults.

## 1. Introduction

Age-related hearing loss is a highly prevalent health concern for people over the age of 40 [1,2]. Indeed, the onset of hearing loss due to aging is normally observed around the age of 40, with a gradual decrease in hearing sensitivity henceforth (e.g., [2]). Ageing is also accompanied by a steady decline in vestibular and touch sensitivity and acuity (for a review, see [3,4,5]) and is also associated with alterations in muscle spindles and their neural pathways, which induces a decrease in the integration of the proprioceptive signals [6]. These alterations promote changes in postural control that reduce its efficiency.

In recent years, the important role of auditory function in postural control has gained general support. Indeed, there is growing evidence linking hearing impairments with negative changes in postural stability (for a review, see [7]). Notably, previous studies have demonstrated that hearing-impaired individuals have reduced static postural control when compared to normal hearing individuals (e.g., [8,9,10,11]), as well as an increased risk of falling (e.g., [7,12,13,14]). Recent evidence indicates a positive association between the severity of hearing impairment and poorer postural stability in older adults (for a systematic review and meta-analysis, see [15]). 

Alterations in auditory input also seem to lead to sensory reweighting for postural control. In this context, sensory reweighting refers to the process of adjusting the contribution of the visual, vestibular, and somatosensory systems toward maintaining postural control when one of these sensory modalities is disturbed [16]. During such a sensory perturbation, a feedback control model allows for an increase in the weighting of a more consistent sensory modality and a simultaneous decrease in the reliance on the disturbed modality in order to better maintain posture [17]. 

The data suggest that hearing loss might have an impact on such sensory reweighting during sensory perturbations. For example, postural control has been examined in congenitally deaf and normal hearing adults using a force platform and the modified clinical test of sensory interaction and balance protocol [18]. The experiment was conducted without auditory cues, namely, without hearing aids in the deaf and with hearing protectors in normal hearing individuals. Using a calculation designed to approximate sensory reliance (see [19]), the data revealed that congenitally deaf individuals relied more on somatosensory information for postural control than normal hearing individuals. These results raise important questions with regards to age-related hearing loss. Indeed, if sensory reweighting is similar in age-related hearing loss (i.e., presbycusis) as in congenital hearing loss, it is likely that it would not effectively mitigate the decrease in postural control. Indeed, an increased reliance on the somatosensory system to maintain posture in this specific population would likely be unhelpful considering that somatosensory processes also decline with healthy ageing. 

In order to further define the role of auditory perception in postural control and to further the understanding of sensory-related mechanisms associated with postural instability in the older adults, the aim of this study was therefore to determine whether sensory reweighting for postural control occurs in older adults with age-related hearing loss. Based on previous investigations on congenitally deaf individuals, we hypothesized that older people with hearing loss would rely more on somatosensory information for postural control than normal hearing individuals.

## 2. Materials and Methods

Seventy-one individuals over the age of 18 took part in the study. Among those, 20 participants were aged between 18 and 25 (13 women and 7 men) and 51 participants were aged over 40 (28 women and 23 men). Participants were recruited using an existing participant pool and through posters placed on bulletin boards at the Université de Montréal. Participants had no history of noise exposure, diabetes, cardiovascular diseases, pulmonary diseases, head trauma, neurological disorders, orthopedic pathology, or musculoskeletal dysfunctions. They also had no history of falls or lower limb surgery within the last 2 years, nor a BMI over 40 kg/m^2^. All participants provided a written informed consent prior to the experiment. They all had normal or corrected-to-normal vision and no history of vestibular disorders such as vertigo. A medication check was also conducted to identify whether any participants’ postural control was negatively impacted by medication. Experimental procedures were approved by the University of Montreal and the Center for Interdisciplinary Research in Rehabilitation of Greater Montreal research ethics boards.

All participants were administered a hearing test by a certified audiologist, including otoscopy, tympanometry, and pure-tone audiometry using air and bone conduction. Hearing threshold levels were assessed with an audiometer (Astera, GN Otometrics, Denmark) using ER-3A earphones and a Radioear B-71 bone oscillator. The frequencies tested by octave bands included 250 Hz to 8 kHz (with the addition of 3 kHz and 6 kHz) for air conduction and 250 Hz to 4000 Hz for bone conduction. A comparison between both types of conduction allowed for the determination of the etiology of the hearing loss. Normal hearing sensitivity was defined as all hearing threshold levels below 15 dB HL, as per the American Speech-Language-Hearing Association (ASHA). After the audiological evaluation, participants were assigned to either the group with or the group without hearing loss.

Mean hearing thresholds (mean of 0.25, 0.5, 1, 2, 3, 4, 6, and 8 kHz) for participants aged 18 to 25 were less than 15 dB HL (mean hearing threshold: 3.41, SD: 3.64). Among participants over the age of 40, only 11 individuals (mean age: 55.82, SD: 7.8) had hearing thresholds of 15 dB HL or less (mean hearing threshold: 10.00 dB HL, SD: 2.96). 

The remaining individuals over 40 (*n* = 40, mean age: 67.35, SD: 9.8) had age-related hearing loss (mean hearing threshold: 43.2 dB HL, SD: 19.70), ranging from mild to severe, which was diagnosed in accordance with standard ISO7029: 2017 [20]. Thirty-seven of the participants with hearing loss were using hearing aids. 

Participants were invited to perform the modified clinical sensory integration in balance test (mCTSIB) on a force platform (Accusway, AMTI, Watertown, MA, USA) at a sampling rate of 50 Hz. Considering the objective of the study, which was to examine the influence of age-related hearing loss on postural control, participants with hearing loss were tested without their hearing aids. As in most studies evaluating static postural control, pink noise (100 Hz–4 kHz) was used to ensure a uniform auditory environment for all participants. The noise was presented at a comfortable level through a speaker placed one meter behind the participant (Sound Source Type 4224, Bruel & Kjaer, Virum, Denmark). The system recorded changes in the center of pressure (CoP) in the anteroposterior and mediolateral axis. Sway area and velocity were derived from those recordings. Sway area represents the surface of the ellipse covering 95% of the CoP points, while sway velocity represents the average speed of the total displacement [18]. The mCTSIB was used as it allows the isolation of the different sensory components (vision, somatosensory, vestibular) for balance [21]. Participants stood under four different postural conditions: (A) eyes open on a firm surface; (B) eyes closed on a firm surface; (C) eyes open on foam; and (D) eyes closed on foam. The foam pad (AIB Balance Foam, AIB, New York, NY, USA) was standardized to a maximum weight of 159 kg. Each trial lasted 60 s. To ensure that each participant provided an optimal postural performance, participants were asked to count backward starting from one thousand as this specific task has been shown to help maintain postural control [22]. Each sensory condition was repeated three times and the median value under each condition was retained. 

A well-known calculation was used to approximate sensory reliance [19]. The possibility of isolating the contribution of a sensory modality is generated by subtracting the condition where all sensory modalities are optimized from the condition with one disturbed sensory modality. As such, the impact of visual information was evaluated by subtracting the sway parameters of condition A (eyes open on the firm surface) from condition B (eyes closed on the firm surface), and the impact of somatosensory information was evaluated by subtracting the sway parameters of condition A (eyes open on the firm surface) from condition C (eyes open on the foam). These two calculations were performed once for sway area and once for sway velocity. 

In order to examine the impact of aging and hearing loss on postural control, we performed a Kruskall–Wallis analysis of variance between the 3 groups (18–25 years without hearing loss; over 40 years without hearing loss; over 40 years with hearing loss) in the 4 postural conditions (condition A: eyes open/surface firm; condition B: eyes closed/surface firm; condition C: eyes open/foam; condition D: eyes closed/foam). Kruskall–Wallis tests were also conducted to reveal any changes in sensory reliance between the three groups. Dunn–Bonferroni post-hoc tests were conducted when appropriate.

## 3. Results

Pre-experimental analysis confirmed the difference with regards to age [H(2) = 48.017, *p* < 0.001, η^2^ = 0.677] and hearing loss [H(2) = 54.344, *p* < 0.001, η^2^ = 0.77] between the groups. More specifically, the analysis confirmed that the age was significantly different between young individuals and the two groups of older individuals (*p* < 0.01) but not significantly different between the two groups of older individuals (*p* = 0.059). The analysis also confirmed that hearing loss was significantly greater in older individuals characterized with hearing loss compared to the two other groups of individuals (*p* < 0.001), whereas hearing loss was not significantly different between the groups characterized as having normal auditory hearing (*p* = 0.310).

**Figure 1:**
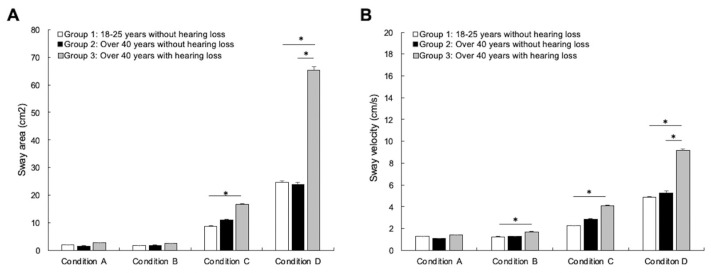
Average sway area (**A**) and sway velocity (**B**) in the four postural conditions (A: eyes open on a firm surface; B: eyes closed on a firm surface; C: eyes open on foam; and D: eyes closed on foam) for the young adults with normal hearing group (white bars, *n* = 20), the older adults with normal hearing group (black bars, *n* = 11), and the older adults with hearing loss group (gray bars, *n* = 40). The data confirm that certain parameters of postural control are reduced in individuals with age-related hearing loss. The error bars represent the standard error of the mean. * *p* < 0.05.

The average sway area and sway velocity in the four postural conditions are illustrated in Figure 1. For sway area, the analysis did not reveal any differences between the groups in condition A [H(2) = 1.1419, *p* = 0.492, η^2^ = 0.013] or condition B [H(2) = 4.289, *p* = 0.117, η^2^ = 0.034]. On the other hand, the analysis revealed significant differences between groups in condition C [H(2) = 18.425, *p* < 0.001, η^2^ = 0.242] and condition D [H(2) = 17.643, *p* < 0.001, η^2^ = 0.23]. Paired comparisons with Bonferroni correction revealed no differences between young adults with normal hearing and older adults with normal hearing in condition C (*p* = 0.182) and condition D (*p* = 1.00). However, the analysis revealed that young adults with normal hearing had significantly less postural sway in condition C (*p* < 0.001) and condition D (*p* < 0.001) compared to older adults with age-related hearing loss (see Figure 1A). The data failed to reveal a significant difference between the two groups of older individuals in condition C (*p* = 0.504) but revealed a significant difference in condition D, as older adults with age-related hearing loss showed significantly more postural sway (*p* = 0.007).

For sway velocity, the analysis revealed no difference between the groups in condition A [H(2) = 5.962, *p* = 0.051, η^2^ = 0.058]. On the other hand, the analysis revealed significant differences between the groups in condition B [H(2) = 7.275, *p* = 0.026, η^2^ = 0.078], condition C [H(2) = 32.382, *p* < 0.001, η^2^ = 0.447], and condition D [H(2) = 19.526, *p* < 0.001, η^2^ = 0.258]. Paired comparisons with Bonferroni correction revealed no differences between young adults and older adults with normal hearing in condition B (*p* = 1.000), condition C (*p* = 0.114), and condition D (*p* = 1.00). The data revealed a significant difference between young individuals and older individuals with age-related hearing loss in condition B (*p* = 0.049), condition C (*p* < 0.001), and condition D (*p* < 0.001), as young adults with normal hearing showed reduced sway velocity compared to older adults with age-related hearing loss (see Figure 1B). The data failed to reveal a significant difference between the two groups of older individuals in condition B (*p* = 0.202) and condition C (*p* = 0.074) but revealed a significant difference in condition D, as older adults with age-related hearing loss showed a significant increase in sway velocity (*p* = 0.012) (Figure 1B). Combined with the analysis for sway area, these results confirm a significant decrease in postural control in participants with hearing loss compared to normal hearing individuals. Such decrease in postural control appears to be independent of aging, as no difference was found between young adults and older individuals with normal hearing.

**Figure 2:**
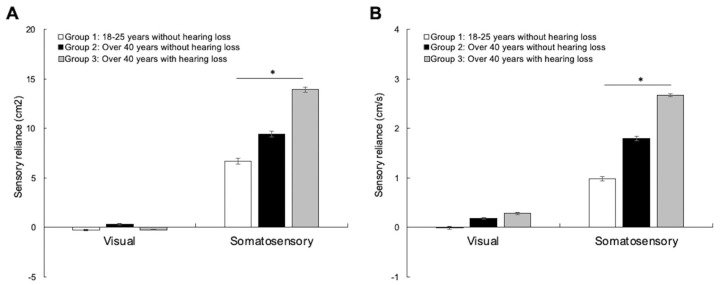
Sensory reliance for sway area (**A**) and sway velocity (**B**) for the young adults with normal hearing group (white bars), the older adults with normal hearing group (black bars), and the older adults with hearing loss group (grey bars). The results suggest an increase in somatosensory reliance for postural control in individuals with age-related hearing loss. The error bars represent the standard error of the mean. * *p* < 0.05.

Sensory reliance for sway area and sway velocity for the three groups are represented in Figure 2. Kruskall–Wallis tests were conducted to reveal any changes in sensory reliance between the three groups. There was no significant difference in visual reliance between the groups (Figure 2A), neither for sway area [H(2) = 1.532, *p* = 0.465, η^2^ = 0.007] nor sway velocity [H(2) = 2.592, *p* = 0.274, η^2^ = 0.009]. However, the analysis revealed significant differences in somatosensory reliance between the groups (Figure 2B), both for sway area [H(2) = 14.692, *p* < 0.001, η^2^ = 0.187] and sway velocity [H(2) = 28.877, *p* < 0.001, η^2^ = 0.395]. Paired comparisons with Bonferroni correction revealed no differences between groups with normal hearing for sway area (*p* = 0.092) or sway velocity (*p* = 0.052). However, the analysis revealed a significant difference between young individuals and older adults with age-related hearing loss for sway area (*p* < 0.001) and sway velocity (*p* < 0.001), suggesting an increase in somatosensory reliance for postural control in older adults with age-related hearing loss. The analysis did not reveal significant differences between the two groups of older individuals for either sway area (*p* = 1.000) or sway velocity (*p* = 0.272). Using Pearson correlational analysis, we were unable to find a relationship between sway area, sway velocity, or sensory weighting in all postural conditions and the severity of hearing loss (*p* > 0.05). There was also no significant effect of gender on these measures (*p* > 0.05). The results suggest that normal hearing individuals, no matter their age, use the different senses similarly to maintain postural control. The results concomitantly suggest that hearing loss can have an impact on such sensory weighting, as older adults with hearing loss make significantly greater use of somatosensory modality compared to individuals with normal hearing.

## 4. Discussion

The aim of this study was to examine possible sensory reweighting for postural control in older adults with and without age-related hearing loss. Consistent with studies suggesting poorer postural control in the elderly and in individuals with hearing loss (e.g., [12,18,23,24,25]), the analyses support the important role of hearing in postural control, which is independent of aging. 

Consistent with previous research on congenital hearing loss [18], our results suggest an increase in somatosensory reliance in older individuals with age-related hearing loss. Considering the general decline in touch sensitivity and acuity (for a review, see [3]), this increased weighting of the somatosensory modality to maintain posture, unfortunately for the individuals, appears to be ineffective in re-establishing better postural control. 

The interpretation of the present results is limited by some elements. Notably, concomitant sensory, perceptual, and motor deficits associated with aging could impact the results. In the present study, the status of other sensory modalities was not examined. To fully confirm the specific impact of age-related hearing loss on sensory reweighting for postural control, one would need to compare the performance of older adults with only an auditory deficit with that of individuals without any kind of sensory impairment. It is possible that older adults with normal hearing have some sort of resistance to an age-related decline of the inner ear. If so, one could wonder if the vestibular system, sharing the same organ, could also be more resistant to age-related decline. As such, future studies should focus on determining the impact of concomitant vestibular dysfunction. Indeed, hearing and vestibular loss have great comorbidity [26] and vestibular dysfunction in adults aged over 40 is highly prevalent [27,28]. However, the impact of vestibular dysfunction may be difficult to determine in the elderly. Even in the eventuality of normal-like peripheral vestibular signals, aging can have an impact on the cortical efficiency with which these signals are used for postural control (for reviews, see [4,5]). Therefore, it might be difficult to precisely estimate the vestibular decline in this population and therefore to fully eliminate a possible impact of this variable on the results. Still, the well-established and effective ways of measuring peripheral vestibular function, as well as comparisons between younger and older adults, could certainly provide indications as to how vestibular function may be deteriorating with healthy ageing.

If concomitant conditions related to aging could indeed have an impact on the results found in participants with age-related hearing loss, other studies support the likelihood of a direct impact of auditory inputs on postural control, in particular, studies using auditory amplification. Indeed, if the deterioration in postural control is specifically linked to the auditory loss, the restoration of the auditory input should have a beneficial impact on the results. Recent studies indeed suggest that hearing aids may improve static posture in people with deafness (for a review, see [29]). These results suggest that the loss of postural control in hearing-impaired individuals is largely specifically linked to the deterioration of the auditory function and not to other variables related to deafness. In future studies, it would also be interesting to examine the impact of hearing aid use on sensory reweighting for postural control in older adults with age-related hearing loss. The data could help determine if improvement is associated with more adequate sensory weighting to maintain balance. Such data could provide an additional argument to encourage early hearing aid fitting in people with hearing loss.

## 5. Conclusions

From a clinical perspective, it is important to note that the risks of falls increase with age (e.g., [30]). Falls are the leading cause of hospitalization among people over the age of 65 and have a significant impact on older adults’ health (WHO, 2021). They are also the leading cause of injury-related mortality in adults aged 65 and older [31]. Hearing loss, of course, is a significant risk factor for falls among older adults [12,32]. Therefore, the current results add to prior work by providing evidence that age-related hearing loss is associated with an increased reliance on somatosensory input and results in increased postural instability in challenging situations. Beyond helping to determine the impact of age-related hearing loss on postural control, the outcomes of this research, along with further investigation, could be used to provide guidelines enhancing the safety of the elderly population during ambulation, particularly in relation to audiological intervention.

## Data Availability

The data presented in this study are available on request from the corresponding author.
